# Zinc Exploitation by Pathogenic Fungi

**DOI:** 10.1371/journal.ppat.1003034

**Published:** 2012-12-20

**Authors:** Duncan Wilson, Francesco Citiulo, Bernhard Hube

**Affiliations:** 1 Department of Microbial Pathogenicity Mechanisms, Leibniz Institute for Natural Product Research and Infection Biology, Hans Knöll Institute (HKI), Jena, Germany; 2 Novartis Vaccines & Diagnostics, Siena, Italy; 3 Center for Sepsis Control and Care, Jena University Hospital, Jena, Germany; 4 Friedrich Schiller University, Jena, Germany; Duke University Medical Center, United States of America

## Introduction

The ability of pathogenic microorganisms to assimilate nutrients from their host environment is one of the most fundamental aspects of infection. To counteract this, hosts attempt to withhold essential micro-nutrients from potentially harmful microbes to limit, or even prevent, their growth. This process is called nutritional immunity. For example, vertebrates, such as humans, express several iron-binding molecules to maintain extremely low free levels of this metal in the body. To overcome this restriction, successful pathogens have evolved sophisticated mechanisms to assimilate iron. These include high affinity transporters, siderophores, and transferrin-, ferritin-, and haem-binding proteins [1], [2]. Indeed, iron acquisition is considered a vital virulence factor for many pathogens. However, nutritional immunity does not begin and end with iron. Vertebrates have also developed mechanisms to sequester other essential metals, such as zinc [3], [4]. The importance of zinc sequestration and the strategies that successful pathogens employ to overcome this has only recently been realized.

## Why Is Zinc So Important?

Zinc is essential for life, with an astonishing 9% of eukaryotic proteins predicted to be zinc metalloproteins [5]. The role of this metal in human health is well-documented [6] and zinc is known to play key roles in both adaptive and innate immunity. However, host zinc sequestration from pathogens, as a means to control microbial growth, is an emerging field [7]. Accompanying this, a growing body of literature is illuminating the role of bacterial zinc acquisition systems in virulence [1]. But what about fungal pathogens? Fungi also rely on zinc for growth, as this metal serves as a cofactor for several enzymes, including superoxide dismutase and alcohol dehydrogenase, along with numerous other proteins, such as transcription factors [8]. Therefore, in order to cause infections, pathogenic fungi must assimilate zinc from their host environment. Here, we will discuss the mechanisms of zinc exploitation by human pathogenic fungal species.

## How Can the Host Sequester Zinc from Potential Invaders?

Although zinc is the second most abundant transition metal in the human body, its spatial distribution is highly dynamic. In mammals, this is largely mediated at the cellular level by zinc transporters. These include ZIPs (Zrt-, Irt-like proteins/solute carrier family 39, SLC39), which deliver zinc into the cytoplasm and ZnTs (zinc transporter), which pump zinc out of the cell or into vesicles [9]. Within the cell, zinc availability is tightly regulated via sequestration within organelles and binding to proteins such as metallothioneins [8]. During acute inflammation, hepatocytes remove zinc from the plasma via ZIP14 [10], reducing availability to extracellular pathogens. On the other hand, ZnT transporters reduce cytoplasmic zinc levels, possibly limiting access to intracellular pathogens; indeed, *Listeria monocytogenes* relies on two zinc uptake systems for intracellular growth [11]. Furthermore, it would appear that ZIP-mediated export of zinc from phagosomes may also be employed to limit the growth of phagocytosed intracellular pathogens [12], [13]. In contrast, it has also been shown that phagocytes attempt to kill intracellular pathogens, such as *Mycobacterium tuberculosis*, by increasing the heavy metal content of intracellular compartments to potentially toxic levels [14], [15]. Therefore, both metal restriction and metal overload represent potential mechanisms to control pathogenic microorganisms.

In addition to cellular import/export, zinc can also be limited via calprotectin, an antimicrobial peptide with zinc (and manganese) chelation properties [3]. Neutrophils (one of the most important antifungal effectors) contain high levels of calprotectin and these phagocytes decorate their NETs (neutrophil extracellular traps) with this potent antimicrobial peptide. Indeed, calprotectin is particularly important for the candidacidal activity of NETs [4].

Intriguingly, *Salmonella* Typhimurium can actually exploit calprotectin-mediated zinc sequestration in the inflamed guts of infected mice, thus out-competing rival commensal species [16]. Nutrient acquisition from this antimicrobial peptide represents a striking example of a pathogen gaining an upper hand in the continuous arms-race with its host. It remains an open question whether fungal pathogens can also exploit this potential zinc source during infection.

## How Do Fungi Obtain Zinc?

**Figure 1:**
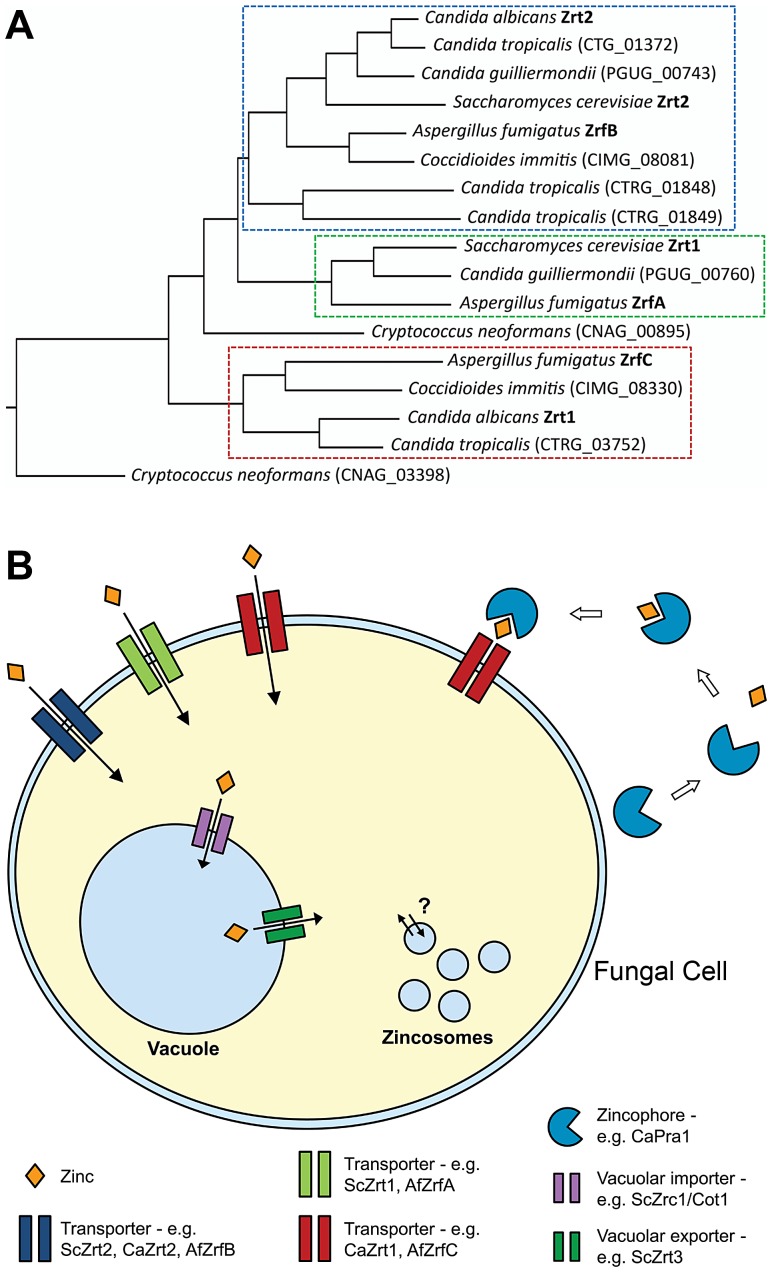
Fungal zinc acquisition. (A) Schematic of zinc transporter phylogeny from selected fungal pathogens; *S. cerevisiae* is included for comparison. Note the presence of three clusters; only transporters of the third class (red dashed box) are encoded syntenically with the zincophore (Pra1). (B) An archetypal fungal cell acquires zinc via cell membrane transporters, intracellular mobilization of stored zinc, and a zincophore scavenging system. Note that the third class of transporter (red) also mediates zincophore reassociation.

A number of studies have investigated the mechanisms of zinc homeostasis in the model yeast *Saccharomyces cerevisiae*. This fungus encodes two plasma membrane transporters, Zrt1 and Zrt2 (Figure 1A and 1B), which are up-regulated by the transcription factor Zap1 and transport zinc into the cell. If cellular levels become too high, these importers are rapidly down-regulated [17].

The fungal vacuole is also extremely important for cellular zinc homeostasis: Cot1/Zrc1 (ZnT type transporters) import zinc into the fungal vacuole, thus protecting the cell from potentially toxic levels of the metal; conversely, if the cell experiences zinc starvation, the zinc pool stored in the vacuole can be mobilized via the vacuolar zinc exporter, Zrt3 (Figure 1B) [18]. Indeed, vacuolar storage and mobilization can significantly buffer the cytosol from both zinc excess and depletion. Unlike bacteria, which express cell membrane-localized zinc efflux systems [19], fungi do not appear to actively export zinc in this manner [17] and rather rely on vacuolar sequestration to detoxify this metal.

The CtpC zinc efflux system of *M. tuberculosis* is required for survival within macrophages, as these immune cells attempt to poison phagocytosed bacteria with potentially toxic levels of zinc [14], [15]. Although it is at present unknown whether phagocytosed fungi also experience zinc toxicity, vacuolar detoxification would represent a promising mechanism for counteracting this [20]. Indeed, orthologues of vacuolar zinc transporters are found in several pathogenic fungi.

As well as counteracting metal toxicity, intra-vacuolar storage can also endow yeasts with an extraordinary capacity to withstand zinc starvation. Simm et al. have demonstrated that the storage capacity of the *S. cerevisiae* vacuole is sufficient for a single mother cell to produce many progeny, even in the absence of zinc uptake [21]. Thus vacuolar zinc storage may have serious implications for virulence studies aimed at elucidating zinc uptake systems in fungal pathogens. Zincosomes represent another potential intracellular zinc store (Figure 1B). Zincosomes are vesicles that contain labile zinc and have been observed in both mammalian and yeast cells. Zincosomes may serve to both detoxify excess zinc and mobilize this metal upon deprivation (analogous to the fungal vacuole); however, the exact nature of these compartments, and the mechanisms by which they function, remain unclear [17].

Zinc acquisition by a pathogenic fungus has been most extensively investigated in *Aspergillus fumigatus* by the Calera group. *A. fumigatus* encodes three zinc transporters (*zrfA*-*C*), all of which are positively regulated by ZafA, the functional orthologue of yeast Zap1 [22], [23]. Although the role of these transporters in *A. fumigatus* pathogenicity has not yet been directly examined, deletion of their transcriptional activator, ZafA, abrogates virulence, suggesting a role for zinc uptake during infection [23]. Similarly, *Candida albicans* Zap1 is required for the expression of zinc transporter encoding genes and plays a crucial role in biofilm formation, an important pathogenicity attribute of this fungus [24]. Therefore, the transcriptional regulation of zinc transporters would appear to be conserved, at least amongst *S. cerevisiae*, *C. albicans*, and *A. fumigatus*.

Phylogenetic studies have identified zinc transporters in numerous other pathogenic fungi [25] and Figure 1A; however, their roles in virulence remain largely unexplored. In addition to transporter-mediated assimilation, zinc can also be scavenged via the zincophore system.

## The Fungal Zincophore System

We recently reported sequestration of host zinc by the major human fungal pathogen *C. albicans* [25]. We hypothesized that, analogous to siderophore-mediated iron acquisition, the fungus might secrete a zinc-binding molecule to scavenge this metal from its environment—we proposed the term “zincophore” for such a scavenger. Indeed, we found that *C. albicans* secretes a zinc-binding protein (Pra1, pH-regulated antigen), which can sequester this metal from the environment and is required for stealing zinc from host cells (Figure 1B). Moreover, Pra1 reassociation with the fungal cell (a prerequisite for a functional zincophore system) was found to be mediated by the plasma membrane zinc transporter (Zrt1), encoded at the same locus as the zincophore. Therefore, Zrt1 of *C. albicans* likely has dual transporter and receptor functions (Figure 1B).

The Calera lab has shown that the Pra1 and Zrt1 orthologues of *A. fumigatus* (Aspf2 and ZrfC, respectively) are also syntenically encoded, regulated by environmental zinc status and required for growth under zinc starvation [22], indicating functional conservation of zinc-acquisition loci between *Candida* and Aspergilli.

Indeed, we found the zincophore locus in both ascomycetes and basidiomycetes, groups of fungi that diverged around half a billion years ago—clearly, this system is not specific to human pathogens. Rather, it appears to be associated with zinc foraging in certain niches of neutral/alkaline pH. In line with this, both characterized systems (in *A. fumigatus* and *C. albicans*) are strongly repressed by acidic pH, even under conditions of zinc limitation [22], [25]. Although the zincophore system is essential for *C. albicans* zinc scavenging during host cell invasion [25], many other human fungal pathogens do not encode Pra1 [25]. These other species must rely on alternative acquisition systems. It is possible that some species rely solely on transporters for zinc uptake. Alternatively, another, convergently evolved, secreted zinc-binding protein may be deployed. Finally, it is possible that some fungi may secrete small molecule zinc chelators to sequester this metal.

The most well characterized zincophore-encoding species, *C. albicans* and *A. fumigatus*, are both notable for aggressive, hypha formation-mediated tissue invasion and inflammation. Indeed, Pra1 is immuno-modulatory [26] and is the major ligand for leukocyte integrin αMβ2 [27]. Similarly, the *A. fumigatus* homologue Aspf2 is a major allergen, which cross-reacts with over 80% of sera from patients suffering from aspergilloma or allergic bronchopulmonary aspergillosis [28].

Therefore, an intriguing possibility is that the loss of the zincophore system by contemporary human fungal pathogens [25] may actually contribute to their capacity to evade certain aspects of immune recognition. Whilst foregoing zincophore-mediated zinc scavenging, yeasts such as *C. glabrata*, *Cryptococcus neoformans* and *Histoplasma capsulatum* may benefit from avoiding unwanted attention from aggressive host immune responses.

In summary, as the scope of nutritional immunity expands beyond iron to encompass other metals, the molecular mechanisms that pathogenic microorganisms deploy to circumvent host metal restriction represents fertile ground for the identification of novel virulence factors.
